# The Role of SOX9 in IGF-II-Mediated Pulmonary Fibrosis

**DOI:** 10.3390/ijms241411234

**Published:** 2023-07-08

**Authors:** Kristy M. Waldrep, Jessalyn I. Rodgers, Sara M. Garrett, Bethany J. Wolf, Carol A. Feghali-Bostwick

**Affiliations:** 1 Department of Medicine, Rheumatology, Medical University of South Carolina, Charleston, SC 29425, USA; waldrepk@musc.edu (K.M.W.); ierardi@musc.edu (J.I.R.); drsaragarrett@gmail.com (S.M.G.); 2 Department of Public Health Sciences, Biostatistics and Bioinformatics, Medical University of South Carolina, Charleston, SC 29425, USA; wolfb@musc.edu

**Keywords:** systemic sclerosis/scleroderma, pulmonary fibrosis, insulin-like growth factor-II (IGF-II), SRY-box transcription factor 9, pulmonary/lung fibroblasts

## Abstract

Pulmonary fibrosis (PF) associated with systemic sclerosis (SSc) results in significant morbidity and mortality. We previously reported that insulin-like growth factor-II (IGF-II) is overexpressed in lung tissues and fibroblasts from SSc patients, and IGF-II fosters fibrosis by upregulating collagen type I, fibronectin, and TGFβ. We now show that IGF-II augments mRNA levels of profibrotic signaling molecules *TGFβ2* (*p* ≤ 0.01) and *TGFβ3* (*p* ≤ 0.05), collagen type III (*p* ≤ 0.01), and the collagen posttranslational modification enzymes *P4HA2* (*p* ≤ 0.05), *P3H2* (*p* ≤ 0.05), *LOX* (*p =* 0.065), *LOXL2* (*p* ≤ 0.05), *LOXL4* (*p* ≤ 0.05) in primary human lung fibroblasts. IGF-II increases protein levels of TGFβ2 (*p* ≤ 0.01), as well as COL3A1, P4HA2, P4Hβ, and LOXL4 (*p ≤* 0.05). In contrast, IGF-II decreases mRNA levels of the collagen degradation enzymes cathepsin (CTS) K, *CTSB*, and *CTSL* and protein levels of CTSK (*p* ≤ 0.05). The SRY-box transcription factor 9 (SOX9) is overexpressed in SSc lung tissues at the mRNA (*p* ≤ 0.05) and protein (*p* ≤ 0.01) levels compared to healthy controls. IGF-II induces SOX9 in lung fibroblasts (*p ≤* 0.05) via the IGF1R/IR hybrid receptor, and SOX9 regulates TGFβ2 (*p* ≤ 0.05), TGFβ3 (*p* ≤ 0.05), COL3A1 (*p* ≤ 0.01), and P4HA2 (*p* ≤ 0.001) downstream of IGF-II. Our results identify a novel IGF-II signaling axis and downstream targets that are regulated in a SOX9-dependent and -independent manner. Our findings provide novel insights on the role of IGF-II in promoting pulmonary fibrosis.

## 1. Introduction

Pulmonary fibrosis (PF) is characterized by abnormal scarring of lung tissue due to overproduction of extracellular matrix (ECM) components [1]. PF is the leading cause of death in patients with systemic sclerosis/scleroderma (SSc) [2,3]. The loss of lung function and limited treatment options result in significant morbidity and mortality for affected patients [4]. Several therapies have been investigated for their ability to modulate dermal fibrosis in SSc. For example, the active metabolite of Selexipag (ACT-333679) demonstrated antifibrotic effects in fibroblasts from SSc skin [5]. However, Nintedanib and tocilizumab are currently the only FDA-approved drugs for the treatment of SSc-PF [6,7,8]. These treatments merely slow the progression of fibrosis and fail to halt or reverse disease [9,10]. Lung transplantation is the only curative option, but it is not feasible at the scale that is needed [4]. Consequently, there is a substantial need for the development of new, effective treatments, a process that ultimately relies on a better understanding of the mechanisms mediating PF. 

SSc-PF pathogenesis involves injury to vascular endothelial cells, autoimmunity, inflammation, and unchecked activation of resident fibroblasts into myofibroblasts [3,11,12,13]. Fibroblasts normally sustain ECM homeostasis, partly by regulating levels of ECM components such as collagen and fibronectin, thus supporting lung structure, mechanical function, and wound repair [13,14]. During fibrosis, normal mechanisms to resolve healing (i.e., myofibroblast dedifferentiation, senescence, or apoptosis) are defective, and wound repair becomes unchecked [12,13,15]. Several signaling molecules exacerbate repair, including transforming growth factor-beta (TGFβ), interleukin 6 (IL-6), and connective tissue growth factor (CTGF) [13]. Previous research by our group identified insulin-like growth factor (IGF)-II as a profibrotic signaling molecule in fibrotic lungs of SSc patients [16,17]. IGF-II is a peptide hormone essential for fetal growth and development, as well as cellular growth, organ maintenance, and metabolism in adults [18]. IGF-II has been implicated in growth syndromes, obesity, diabetes, polycystic ovarian syndrome (PCOS), and cancer [18,19,20]. In humans, IGF-II is the most prevalent hormone of the IGF system, which includes IGF-I, insulin, their receptors, and IGF-binding proteins (IGFBPs) [19,20,21]. IGF-II may bind to four receptors, the insulin receptor (IR), the IGF-1 receptor (IGF1R), a hybrid of these two (IGF1R/IR), and the IGF-2 receptor (IGF-2R). Only the IR, IGF1R, and IGF1R/IR receptors result in downstream signaling [18]. 

We previously reported elevated IGF-II levels in lung tissues and fibroblasts from SSc patients [16]. IGF-II expression is also increased in tissues and fibroblasts from patients with idiopathic pulmonary fibrosis (IPF), a similar but distinct disease, as reported in single-cell RNA sequencing studies accessible on the IPF Cell Atlas portal [22,23,24,25]. IGF-II stimulation of normal lung (NL) and SSc fibroblasts increased collagen type I and fibronectin through the JNK and PI3K signaling pathways and the hybrid IGF1R/IR receptor [16,17]. Furthermore, IGF-II increased TGFβ expression, activated fibroblasts into myofibroblasts, and altered levels of matrix metalloproteinases (MMPs), which are ECM-degrading enzymes, and tissue inhibitors of metalloproteinases (TIMPs), which inhibit MMP function [17]. These findings established the role of IGF-II as a profibrotic factor in PF. 

The mechanism by which IGF-II mediates fibrosis is not fully understood. Therefore, our goal was to provide mechanistic insights into the role of IGF-II in PF using primary human lung fibroblasts and tissues from healthy individuals (NL) and SSc patients. Based on our recent characterization of NL and SSc lung fibroblast transcriptomic signatures [26,27], we identified novel targets downstream of IGF-II that include collagen type III (COL3A1), several collagen posttranslational modification enzymes, and collagen degradation enzymes. In addition, we investigated SRY-box transcription factor 9 (SOX9) as a mediator of the IGF-II fibrotic response. SOX9 regulates the ECM in chondrogenesis, cancer, growth and development, and fibrosis [28,29,30,31]. In this study, IGF-II increased expression of SOX9 in human lung tissues and fibroblasts, and SOX9 mediated the IGF-II regulation of transforming growth factor beta 2 (TGFβ2) and 3 (TGFβ3), COL3A1, and prolyl 4-hydroxylase subunit alpha 2 (P4HA2) in lung fibroblasts. Our data identify a novel IGF-II-SOX9 pathway which can be leveraged for the development of new therapeutic interventions for PF. 

## 2. Results

### 2.1. IGF-II Promotes Fibrosis in Normal Human Lung Fibroblasts and Tissues from Different Donors 

**Figure 1:**
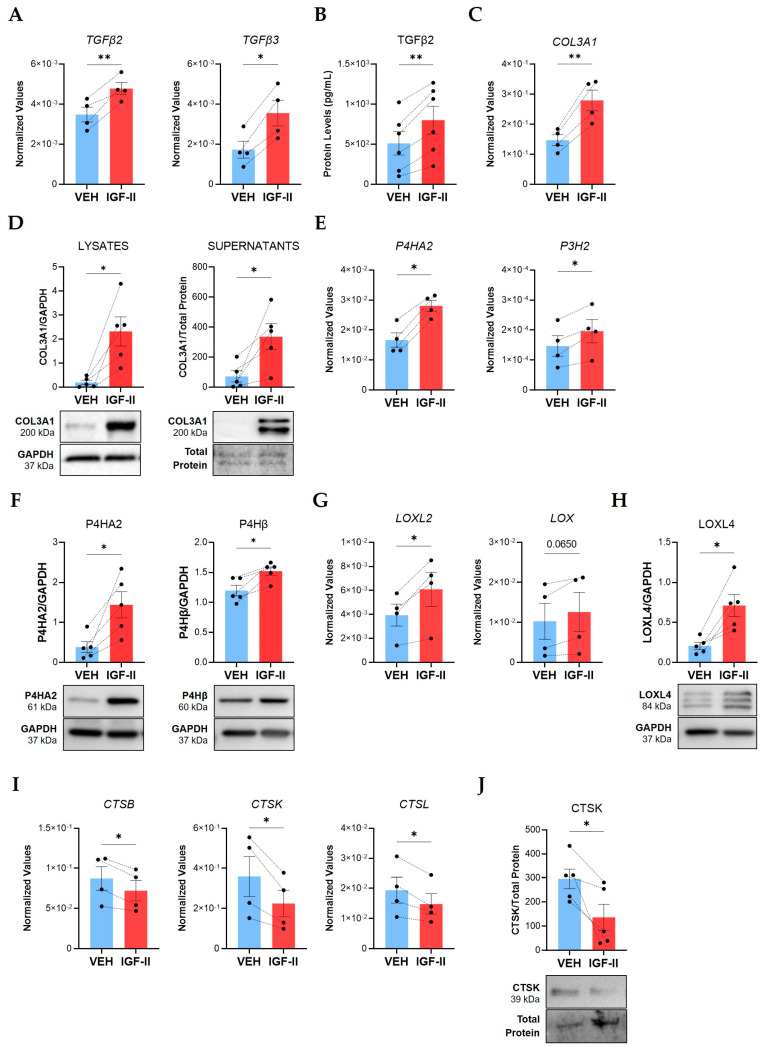
IGF-II promotes fibrosis in normal human lung fibroblasts from multiple donors. Human NL fibroblasts were stimulated with recombinant human IGF-II (200 ng/mL) and PBS/vehicle (VEH) for 24 and 48 h. (**A**) Transcript levels of *TGFβ2* and *TGFβ3* measured by qPCR in fibroblasts after 24 h stimulation (n = 4). (**B**) TGFβ2 concentrations measured by ELISA in 48 h supernatants (n = 6). (**C**) Transcript levels of *COL3A1* as described in panel A. (**D**) Quantified immunoblots of COL3A1 in lysates (24 h) and supernatants (48 h) (n = 5). (**E**) *P4HA2* and *P3H2* transcript levels as described in panel A. (**F**) Quantified P4HA2 and P4Hβ protein levels in 48 h lysates (n = 5). (**G**) *LOXL2* and *LOX* transcript levels as described in panel A. (**H**) LOXL4 levels as described in panel F. (**I**) Transcript levels of *CTSB, CTSK,* and *CTSL* as described in panel A. (**J**) Quantified immunoblot of CTSK in 48 h supernatants (n = 5). The dashed lines indicate pairing between cell lines from the same donor. Raw 2^−ΔCT^ values are graphed for qPCR data after normalization to the housekeeping genes *GAPDH* or *B2M*. Raw densitometry values determined by ImageJ software version 1.53k after normalization to GAPDH are plotted for immunoblot quantification. Paired Student’s *t*-test, * *p* ≤ 0.05, ** *p* ≤ 0.01. Mean ± SEM.

We identified fibrosis-associated targets modulated by IGF-II in human NL fibroblasts and tissues in organ culture. Consistent with our prior report, stimulation with recombinant human IGF-II for 24 h increased transcript levels of *TGFβ2* (*p ≤* 0.01) and *TGFβ3* (*p ≤* 0.05) in NL fibroblasts from additional donors (Figure 1A) [17]. We expanded these findings by measuring TGFβ2 and TGFβ3 protein levels in NL fibroblast supernatants. TGFβ3 protein levels were below the minimum detection range of the available commercial ELISA kits; however, IGF-II significantly increased TGFβ2 protein levels (*p ≤* 0.01) 48 h post-stimulation (Figure 1B). This suggested that IGF-II induces the secretion of TGFβ2, a profibrotic cytokine, into the extracellular space of fibroblasts, thus amplifying fibrotic signaling. We then tested the effects of IGF-II on NL tissue cores in organ culture. IGF-II significantly increased *TGFβ3* transcript levels (*p ≤* 0.01) after 48 h (Figure S1A). *TGFβ2* mRNA levels showed an increasing trend that was not statistically significant, possibly due to masking of the fibroblast response by other cell types (Figure S1B). IGF-II thus promoted fibrosis in human NL fibroblasts and tissues by upregulating the potent profibrotic factors TGFβ2 and TGFβ3. 

We previously demonstrated that IGF-II upregulated collagen type I (COL1A1) and fibronectin in SSc and NL fibroblasts [16,17]. We now show that IGF-II significantly elevated mRNA (*p ≤* 0.01, Figure 1C) and intracellular protein levels (*p ≤* 0.05, Figure 1D) of COL3A1 in NL fibroblasts after 24 h. Extracellular COL3A1 increased in fibroblast supernatants after 48 h (*p ≤* 0.05), indicating IGF-II increases expression and secretion of COL3A1 into the ECM. Likewise, IGF-II significantly increased transcript (*p ≤* 0.05) and protein (*p ≤* 0.05) levels of COL3A1 in NL tissues in organ culture (Figure S1C,D). These findings suggest that IGF-II promotes COL3A1 production in primary lung fibroblasts and in lung tissues. 

Collagen production requires numerous enzymes to ensure proper folding, stabilization, and incorporation into the ECM [32]. We speculated that IGF-II may additionally regulate expression of enzymes responsible for collagen posttranslational modification (PTM), such as prolyl hydroxylases and lysyl oxidases. Indeed, IGF-II stimulation of NL fibroblasts for 24 h significantly increased *P4HA2* (*p* ≤ 0.05) and prolyl 3-hydroxylase 2 (*P3H2*, *p ≤* 0.05) transcript levels (Figure 1E). Accordingly, IGF-II significantly increased P4HA2 (*p ≤* 0.05) and prolyl 4-hydroxylase subunit beta (P4Hβ, *p ≤* 0.05) protein levels in fibroblast lysates (Figure 1F). The lysyl oxidase (LOX) family of enzymes was also regulated by IGF-II, with significantly increased lysyl oxidase-like 2 (*LOXL2*, *p ≤* 0.05) and a trending increase in lysyl oxidase (*LOX*, *p =* 0.065) transcript levels in fibroblasts 24 h post-stimulation (Figure 1G). Furthermore, LOXL4 protein levels significantly increased (*p ≤* 0.05) in fibroblasts after 48 h of stimulation (Figure 1H). In addition to upregulating collagen levels, this data suggests that IGF-II further promotes fibrosis by upregulating the enzymes necessary for collagen processing, stabilization, and incorporation into the ECM. 

Sustained deposition of collagen in fibrosis may involve decreased expression or inactivation of collagen degradation enzymes [33]. Cathepsins and MMPs are the predominant ECM-degrading enzymes in tissue [34]. We previously investigated MMPs and their inhibitors, TIMPs, in IGF-II-mediated fibrosis [17]. To further examine the role of IGF-II in collagen breakdown, we examined other collagenolytic enzymes, specifically cathepsins. IGF-II decreased fibroblast mRNA levels of cathepsins B (*CTSB*), K (*CTSK*), and L (*CTSL*) at 24 h (*p ≤* 0.05, Figure 1I). The 39-kDa zymogen form of CTSK decreased (*p ≤* 0.05) in 48 h fibroblast supernatants following IGF-II stimulation (Figure 1J). This data implies that downregulation of ECM degradation constitutes yet another mechanism by which IGF-II promotes fibrosis. 

Thus, our data further defines an enhanced profibrotic environment initiated by IGF-II. IGF-II increases expression of profibrotic signaling molecules (TGFβ2, TGFβ3), collagen (COL3A1), and collagen PTM enzymes (P4HA2, P3H2, P4Hβ, LOX, LOXL2, LOXL4), while decreasing expression of collagenolytic cathepsins (CTSB, CTSK, CTSL).

### 2.2. SOX9 Is Overexpressed in SSc Lung Tissues 

**Figure 2:**
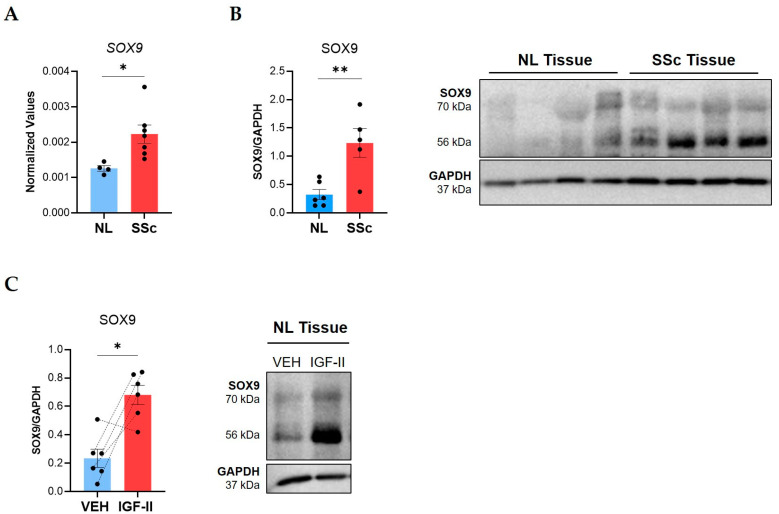
SOX9 is overexpressed in SSc compared to control lung tissues and is induced by IGF-II in human lung tissue cores. Steady-state SOX9 levels were analyzed in lung tissue explants from SSc patients and healthy donors (NL). (**A**) Raw 2^−ΔCT^ qPCR values for *SOX9* normalized to *B2M* in NL (n = 4) and SSc lung (n = 7) tissues. (**B**) Immunoblot quantification of SOX9 bands (56 and 70 kDa) in NL (n = 6) and SSc (n = 5) lung tissues with a representative immunoblot and graphical presentation of data. (**C**) Immunoblot quantification of SOX9 bands (56 and 70 kDa) in NL tissues treated with PBS/vehicle (VEH) or IGF-II (200 ng/mL) for 24 h with a representative blot (n = 6). The dashed lines indicate pairing between cell lines from the same donor. Steady-state SOX9 statistics: unpaired Student’s *t*-test. Induced SOX9 statistics: paired Student’s *t*-test, * *p* ≤ 0.05, ** *p* ≤ 0.01. Mean ± SEM.

Elevated *SOX9* expression has been reported in single-cell RNA sequencing studies of IPF fibroblasts and tissues [23,35] and at the protein level in IPF fibroblasts and tissues [36]. Therefore, we investigated SOX9 involvement in SSc-PF and measured SOX9 levels in lung tissues from SSc patients and controls. Steady-state *SOX9* mRNA levels were significantly elevated (*p ≤* 0.05) in lung tissues from SSc patients (Figure 2A). SSc tissues also had significantly elevated levels of SOX9 protein (*p ≤* 0.01) at 56 kDa, the predicted molecular weight [37], and 70 kDa, a posttranslationally modified form [38] (Figure 2B). 

Given the parallel increase in SOX9 and IGF-II in SSc lung [16], we investigated if IGF-II regulated SOX9 expression in tissues. In NL tissue cores maintained in organ culture, IGF-II stimulation for 24 h significantly increased levels of the 56 kDa and 70 kDa SOX9 forms (*p ≤* 0.05), demonstrating that IGF-II upregulates SOX9 (Figure 2C).

### 2.3. SOX9 Is an Immediate Early Gene Activated by IGF-II through the IGF-1R/IR Hybrid Receptor in Human Lung Fibroblasts

**Figure 3:**
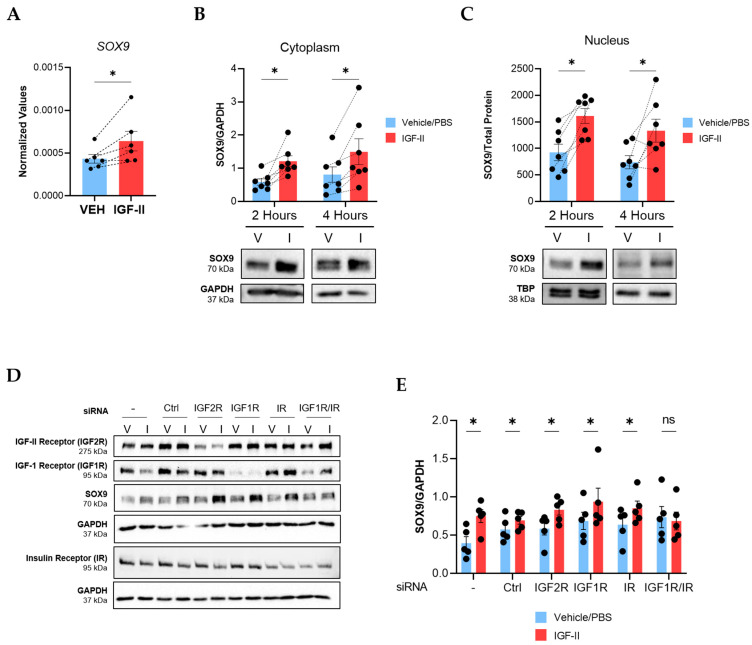
IGF-II induces SOX9 expression in human NL fibroblasts via the hybrid IGF-1R/IR receptor. NL fibroblasts were stimulated with recombinant human IGF-II (200 ng/mL) or vehicle control (PBS) and SOX9 levels were measured. (**A**) qPCR analysis of *SOX9* normalized to *B2M* in NL fibroblasts 1 h post-stimulation (n = 6). Paired Student’s *t*-Test was performed on the raw 2^−ΔCT^ values. (**B**) Immunoblot quantification of cytoplasmic SOX9 after 2 h (n = 7 independent experiments using fibroblasts from six donors) and 4 h of IGF-II stimulation (n = 7 independent experiments using fibroblasts from five donors). Raw densitometry values were normalized to GAPDH. (**C**) Quantification of nuclear SOX9 after 2 h and 4 h in the same fibroblasts as panel B. Raw densitometry values were normalized to total protein concentrations. TBP is also shown as a control. Data were analyzed with repeated measures two-way ANOVA and Tukey’s post-hoc test. (**D**) Representative immunoblots of SOX9 in fibroblast lysates after transfection with scrambled control and receptor-specific siRNA and stimulation with vehicle or IGF-II for 4 h (n = 5). (−): non-transfected control; Ctrl: non-targeting siRNA control; V: vehicle/PBS; I: IGF-II. (**E**) Quantification of immunoblots in panel D. SOX9 raw densitometry values were normalized to GAPDH. Paired Student’s *t*-test, ns = not significant, * *p* ≤ 0.05. Mean ± SEM.

To define the kinetics of SOX9 induction by IGF-II, we performed a time course experiment using human NL fibroblasts from different donors and analyzed *SOX9* mRNA levels by qPCR (Figure S2A). IGF-II stimulation induced *SOX9* as early as 1 h after stimulation (*p ≤* 0.05, Figure 3A) and decreased *SOX9* levels by 24 h (*p* = 0.055, Figure S2A). Nuclear translocation is required for transcription factors to regulate gene expression; therefore, we assessed SOX9 protein levels and subcellular localization in IGF-II-treated fibroblasts. IGF-II increased SOX9 protein levels in the cytoplasm (Figure 3B) and nucleus (Figure 3C) of NL fibroblasts after 2 and 4 h of stimulation (*p ≤* 0.05). The induction was sustained in whole-cell fibroblast lysates after IGF-II stimulation for 24 (*p* = 0.08) and 48 h (*p* ≤ 0.05, Figure S2B). Thus, IGF-II increased expression and nuclear translocation of SOX9 in NL fibroblasts.

We next investigated the receptors that mediate the SOX9 induction downstream of IGF-II. We silenced IGF-2R, IGF1R, IR, and the IGF1R/IR hybrid using small interfering RNA (siRNA) in NL fibroblasts prior to stimulating the cells with IGF-II. Successful knockdown of each receptor was confirmed (Figure 3D and Figure S2C). Only dual silencing of IGF1R and IR prevented the SOX9 induction by IGF-II, implicating the hybrid receptor in IGF-II regulation of SOX9 (Figure 3D,E). IGF-2R internalizes IGF-II and targets it for lysosomal degradation [20]. We therefore confirmed that silencing the IGF1R/IR hybrid receptor did not impact IGF2R levels (Figure S2C), suggesting that loss of SOX9 induction in cells with silenced hybrid receptors was not due to increased uptake and degradation of IGF-II. This data further highlights the importance of the IGF1R/IR hybrid receptor in IGF-II-mediated fibrosis and the regulation of SOX9.

### 2.4. SOX9 Mediates IGF-II Induction of TGFβ2, TGFβ3, COL3A1, and P4HA2 in NL Fibroblasts

**Figure 4:**
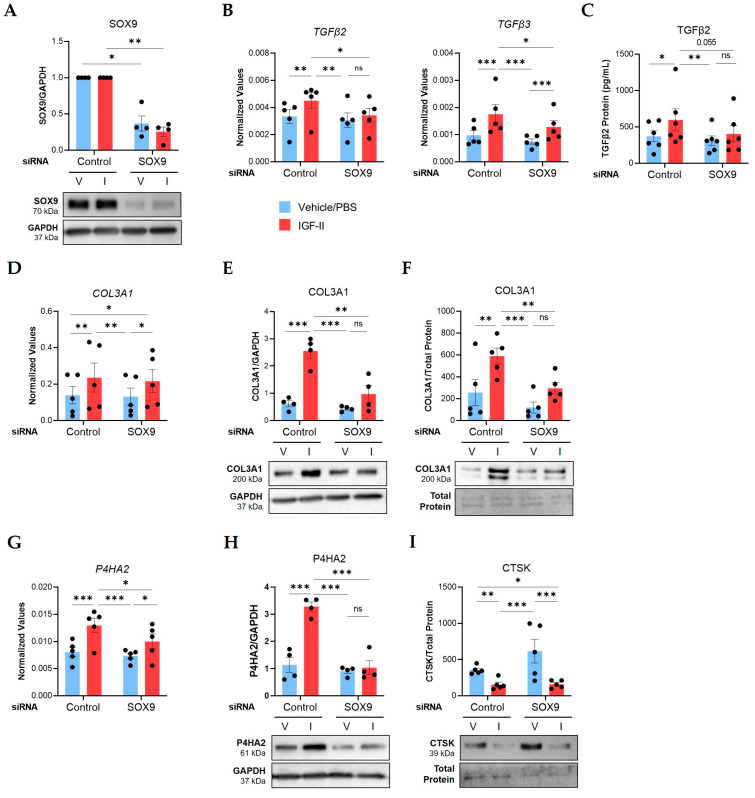
SOX9 mediates IGF-II regulation of TGFβ2, TGFβ3, COL3A1, and P4HA2 in normal human lung fibroblasts. NL fibroblasts from different donors were transfected with non-targeting control or SOX9-specific siRNA before stimulation with vehicle/PBS (V) and IGF-II (I) for 24 or 48 h. (**A**) SOX9 protein levels in lysates at 24 h (n = 4). Fold change values are graphed with the results of repeated measures ANOVA test between the normalized raw densitometry value of control and SOX9-silenced samples respective to treatment. (**B**) Transcript levels of *TGFβ2* and *TGFβ3* at 24 h measured by qPCR (n = 5). (**C**) Protein concentrations of TGFβ2 measured by ELISA in supernatants at 48 h (n = 6 independent experiments in fibroblasts from four donors). (**D**) Transcript levels of *COL3A1* as described for panel B. (**E**) Quantified immunoblot of COL3A1 normalized to GAPDH in lysates at 24 h (n = 4). (**F**) COL3A1 levels in supernatants at 48 h (n = 5) after normalization to total protein concentrations measured by the Bradford assay. (**G**) Transcript levels of *P4HA2* as described for panel B. (**H**) P4HA2 protein levels as described for panel E. (**I**) CTSK levels in supernatants as described for panel F. Raw 2^−ΔCT^ values are graphed for qPCR data after normalization to the housekeeping genes *GAPDH* or *B2M.* Raw densitometry values determined by ImageJ software version 1.53k are plotted for immunoblot quantification. Repeated Measures ANOVA with Tukey’s post-hoc test, ns = not significant, * *p* ≤ 0.05, ** *p* ≤ 0.01, *** *p* ≤ 0.001. Mean ± SEM.

To investigate the role of SOX9 in the IGF-II fibrotic response, we silenced it with siRNA in human NL fibroblasts from multiple donors prior to IGF-II stimulation for 24 or 48 h. Transfection efficiency was confirmed via qPCR and immunoblotting. *SOX9* transcript levels were significantly downregulated in SOX9-silenced (siSOX9) fibroblasts compared to fibroblasts that received non-targeting control siRNA (*p* ≤ 0.05, Figure S3A). SOX9 protein levels were also significantly reduced in SOX9-silenced fibroblasts after 24 h (*p* ≤ 0.01; Figure 4A) and 48 h (*p* ≤ 0.0001; Figure S3B) of IGF-II stimulation. 

Silencing SOX9 abrogated the IGF-II induction of *TGFβ2* (*p* ≤ 0.05) and *TGFβ3* (*p* ≤ 0.05) mRNA levels 24 h post-stimulation (Figure 4B) and reduced secreted TGFβ2 levels (*p* = 0.055) in supernatants after 48 h (Figure 4C). Silencing SOX9 did not change IGF-II regulation of *COL3A1* mRNA levels (Figure 4D); however, it significantly decreased protein levels (*p* ≤ 0.01) in lysates (Figure 4E) and supernatants (Figure 4F). The IGF-II induction of P4HA2 was also inhibited by SOX9 silencing at the mRNA (*p* ≤ 0.05, Figure 4G) and protein levels (*p* ≤ 0.001, Figure 4H; Figure S3C).

We measured other IGF-II-regulated targets after SOX9 silencing in NL fibroblasts. The IGF-II induction of *COL1A1*, *ACTA2*, and *LOXL2* mRNA levels was not changed by SOX9 silencing (Figure S3D). Similarly, SOX9 silencing did not alter the IGF-II regulation of *CTSK*, *CTSL,* and *CTSB* mRNA levels (Figure S3D) or LOXL4 protein (Figure S3E). SOX9 silencing also did not alter secreted CTSK protein levels (Figure 4I). Together, our data identified four profibrotic targets regulated by SOX9 downstream of IGF-II in NL fibroblasts, TGFβ2, TGFβ3, COL3A1, and P4HA2, as well as SOX9-independent targets.

## 3. Discussion

PF is a devastating complication of SSc and IPF. Approved medications reduce disease progression but do not stop or reverse fibrosis [15]. An improved understanding of the mechanisms mediating PF is essential for developing effective treatments. Current drugs target receptor tyrosine kinases (nintedanib), interleukin-6 (tocilizumab), and TGFβ signaling (pirfenidone) [7,39,40]. Our laboratory identified IGF-II as a profibrotic factor overexpressed in fibrotic lung tissues and primary fibroblasts derived from them [16,17]. Understanding the mechanisms mediating IGF-II-induced fibrosis is essential for targeting IGF-II for the treatment of PF. 

Our data show that IGF-II promotes fibrosis in NL fibroblasts and tissues by (a) increasing the expression of profibrotic signaling molecules *TGFβ2* and *TGFβ3* and the secretion of TGFβ2, (b) increasing expression and secretion of the ECM component COL3A1, (c) increasing expression of collagen posttranslational modification (PTM) enzymes P4HA2, P4Hβ, P3H2, LOX, LOXL2, and LOXL4, and (d) decreasing expression of collagen degradation enzymes CTSB, CTSK, and CTSL. We also demonstrate that levels of the transcription factor SOX9 are increased in SSc lung tissues, and IGF-II increases SOX9 levels in lung tissues and fibroblasts. Furthermore, SOX9 is an immediate early gene activated by IGF-II through the IGF-1R/IR hybrid receptor in NL fibroblasts and a mediator of the IGF-II induction of TGFβ2, TGFβ3, COL3A1, and P4HA2. 

### 3.1. The IGF System in Pulmonary Fibrosis 

The IGF system manages multiple physiological processes in humans, including fetal and postnatal growth, neural plasticity, cellular proliferation, and metabolism [41]. This regulation occurs as ligands (IGF-I, IGF-II, or insulin) bind to cellular surface receptors (IR, IGF1R, IGF1R/IR, or IGF-2R) and initiate intracellular signaling that dictates the behavior of target cells. Several IGF members have been implicated in PF, including IGF-I, IGF binding proteins (IGFBP), and IGF-II to a limited extent [42,43,44,45,46,47]. Specifically, IGF-I was shown to induce *aSMA*, *Col1a1*, and *Col3a1* expression in murine lung fibroblasts [42]. Blocking or deleting the IGF-1 receptor (IGF1R) attenuated fibrosis in bleomycin-treated mice [42,43]. In addition, fibrotic lungs (IPF and SSc) have elevated expression of several IGFBPs, including IGFBP2, -3, -5, and -7 [44,45,46,47]. These proteins bind IGF-I and IGF-II to modulate signaling, but also act independently of IGFs. For example, IGFBP5 promotes ECM production and profibrotic signaling in an IGF-I-independent manner [45,48,49,50,51,52]. IGF-II is thus a component of a system-wide dysregulation of IGF signaling that contributes to PF pathology. 

Our group demonstrated that IGF-II is a profibrotic signaling molecule in PF. We found that IGF-II increases TGFβ mRNA levels and signaling, skews TIMP:MMP ratios, activates fibroblasts into myofibroblasts, and increases collagen type I and fibronectin levels in lung fibroblasts and tissues [16,17]. In this study, we sought to advance our understanding of the IGF-II fibrotic mechanism in primary human fibroblasts given that fibroblasts are the main effectors of fibrosis [13]. Our goal was to identify downstream targets of IGF-II to facilitate therapeutic targeting.

IGF-II stimulation increased COL3A1 expression in lung fibroblasts and tissues. It also increased expression of the profibrotic cytokines TGFβ2 and TGFβ3, suggesting that IGF-II intensifies fibrotic signaling. These results align with a study that linked TGFβ and IGF-II signaling to COL3A1 secretion during cell-to-cell interactions between smooth muscle cells (SMCs) and adventitial fibroblasts [53]. Stimulation of SMCs with TGFβ increased secretion of IGF-II and CTGF into supernatants, and rat adventitial fibroblasts responded to these supernatants by upregulating secretion of COL3A1 [53]. This was confirmed in separate experiments by stimulating fibroblasts with recombinant IGF-II and CTGF [53], supporting that IGF-II, TGFβ, and COL3A1 are involved in the same profibrotic pathway and suggesting a positive feedback loop between IGF-II and TGFβ. The IGF-II induction of TGFβ2 and TGFβ3 may render the pathway susceptible to therapeutic intervention with a TGFβ2/TGFβ3-specific antibody, which has recently been investigated, [54] or dual antagonism of IGF-II and TGFβ.

A novel finding in our study is that IGF-II upregulated collagen PTM enzymes. Collagen biosynthesis relies on numerous enzymes to stabilize and process collagen proteins for secretion and incorporation into the ECM [32]. Fibroblasts thus require sufficient levels of collagen PTM enzymes to support excess collagen production during fibrosis [32]. Prolyl-4-hydroxylase (P4H) catalyzes the addition of hydroxyl groups at C4 positions on collagen proline residues [32,55]. This provides thermal and molecular stability to collagen chains, increasing the likelihood of ECM deposition [32,55]. In our study, IGF-II significantly increased the expression of the alpha (P4HA2) and beta (P4Hβ) subunits of P4H, an observation that has not been previously reported. Interestingly, P4HA2 is a catalytic unit of the enzyme, while P4Hβ (protein disulfide isomerase/PDI) converts P4HA2 into its active, soluble form [32,55]. Thus, the IGF-II-mediated upregulation of both subunits suggests increased availability of active P4H in fibroblasts for collagen stabilization. This coincides with studies linking P4H levels to fibroses of the atrium, liver, skin, and lungs [56,57,58,59,60,61]. In SSc, dermal fibroblasts overexpressed P4H [59], and SSc skin tissues showed elevated proline hydroxylation activity [61]. Furthermore, SSc lung fibroblasts from African American patients exhibited increased P4HA2 levels [26]. Our current data shows that IGF-II is an upstream regulator of P4H levels in lung fibroblasts. P4H is considered a significant clinical target in cancer and fibrosis, but P4H inhibitors have not advanced beyond preclinical studies [32,58,62]. Our study suggests that developing new P4H inhibitors may benefit patients with PF. 

The lysyl oxidase protein family, which includes LOX and LOXL1-4 [63], was upregulated by IGF-II in human NL fibroblasts. Specifically, *LOX*, *LOXL2*, and LOXL4 levels were increased by IGF-II. This concurs with IGF-II promoting a fibrotic phenotype as all three of these enzymes are overexpressed in IPF [64]. LOX and LOXL2 are additionally overexpressed in SSc lungs [65,66], and LOXL4 overexpression has been reported in SSc dermal fibroblasts [67]. These enzymes allude to the IGF-II fibrotic mechanism as LOX and LOXL1-4 crosslink collagen fibers in the ECM [68]; therefore, upregulation of these enzymes may represent a means by which IGF-II increases thickness and rigidity of lung tissue during fibrosis. Furthermore, LOX and LOXL2 can regulate cell signaling pathways and act as profibrotic factors [68]. Human lung fibroblasts and tissues stimulated with LOX upregulated COL1A1, fibronectin, and IL-6 [65]. Similarly, LOXL2 regulated COL1A1 levels and activated murine and human fibroblasts into myofibroblasts [64,69]. In primary human cardiac fibroblasts, LOXL2 regulated TGFβ2 levels, downstream fibrotic signaling, and *COL1A1*, *FN1* and *α-SMA* levels [70]. The IGF-II induction of LOX proteins suggests increased collagen crosslinking in the ECM and increased profibrotic signaling via LOX and LOXL2. 

Our data also showed that IGF-II downregulated the expression of multiple cysteine cathepsins (CTSB, CTSK, CTSL). Cathepsins degrade collagen via intracellular lysosomal degradation and secretion into the ECM [71]. Decreased CTSL expression has been reported in SSc and IPF lung tissues and fibroblasts, and TGFβ was shown to downregulate *CTSL* in fibroblasts [27,47]. Our data showing that IGF-II decreases *CTSL* expression in NL fibroblasts suggest that IGF-II limits the availability of this enzyme to reduce collagen breakdown, promoting collagen accumulation. 

We confirmed that IGF-II decreased secreted levels of CTSK in human NL fibroblasts. We predominantly detected the 37-kDa pro-form/zymogen as opposed to the active mature form (24 kDa) in fibroblast supernatants [72]. This is consistent with reports that cathepsin zymogens are secreted into the extracellular space given their stability at a neutral pH compared to their active forms [34,71]. In skin fibroblasts, cathepsin K was shown to localize to lysosomes and degrade collagen types I and IV, and TGFβ decreased cathepsin K expression [73]. CTSK keeps ECM levels in check during normal dermal scarring, and skin tissues with sclerotic morphea exhibited decreased expression of CTSK [74]. Thus, the reduction of CTSK by IGF-II may further support fibrosis by reducing collagen degradation. Restoring levels of cathepsins in fibrosis using enzyme replacement therapy, which has been explored for cathepsin D, may be a viable therapeutic strategy [71,75].

Collectively, our data validate IGF-II as a profibrotic signaling factor in primary lung fibroblasts and lung tissues. IGF-II controls multiple factors of the fibrotic response, including levels of profibrotic signaling molecules (TGFβ2, TGFβ3, LOX, LOXL2), collagen (COL3A1), collagen posttranslational modification enzymes (P4HA2, P4Hβ, P3H2, LOX, LOXL2, LOXL4), and collagen degradation enzymes (CTSL, CTSK, CTSB). IGF-II modifies expression of these targets in a way that promotes a fibrotic phenotype by tipping the balance in favor of fibrosis.

### 3.2. SOX9 in Pulmonary Fibrosis 

Our data showed for the first time that the transcription factor SOX9 is overexpressed in lung tissues from SSc patients compared to normal controls. This extends findings from single-cell RNA sequencing studies reporting increased *SOX9* levels in IPF fibroblasts [25,35,76] and a fibrotic subpopulation of KRT5^-^/KRT17^+^ epithelial cells [23]. Elevated SOX9 levels have also been reported in IPF fibroblasts and tissues, as well as murine fibroblasts in a TGF-α-induced model of PF [36]. Our observed upregulation of SOX9 in SSc lung thus highlights its significance to PF and extends the emerging literature implicating SOX9 in multiple types of fibrosis, including tracheal, liver, cardiac, kidney, and lung [36,77,78,79,80,81]. A recent RNA sequencing analysis of skin biopsies and peripheral blood mononuclear cells from SSc patients identified the non-coding RNA *SOX9-AS1* as a central node in a network of genes correlated with clinical features of SSc, including modified Rodnan skin thickness score, local skin score, forced vital capacity and diffusing capacity for carbon monoxide [82]. SOX9-AS1 increased SOX9 levels by sponging miR-5590-3p [83]. SOX9-AS1 dysregulation in SSc skin may represent one mechanism by which SOX9 levels are elevated in SSc tissues, and IGF-II induction of SOX9 expression provides yet another mechanism. Together with our data, the findings emphasize the important role of SOX9 in SSc and PF. 

To our knowledge, this study is the first report of IGF-II inducing SOX9 expression in primary human lung fibroblasts. Previous reports have examined the effect of IGF-I on SOX9. For example, IGF-I was shown to induce *SOX9* expression in primary human fetal chondrocytes [84] and human adipose-derived mesenchymal cells [85]. This was not the case in IPF fibroblasts, however, as Gajjala et al. did not observe an effect of IGF-I on SOX9 levels [36]. Rather, transforming growth factor alpha (TGF-α), bone morphogenic protein 2 (BMP2), and CTGF induced SOX9 [36]. We show that SOX9 is an early response gene downstream of IGF-II, suggesting that SOX9 is an early mediator of the IGF-II-fibrotic pathway. This is consistent with a study of human chondrocytes where IGF-II increased *SOX9* mRNA levels 5 h post-stimulation [86]. 

The IGF-II-mediated induction of SOX9 in lung fibroblasts occurred via the hybrid IGF1R/IR receptor. IR and IGF1R may exist as homodimers or heterodimers (hybrids) on cellular surfaces due to significant homology between the monomers [87,88]. Both are receptor tyrosine kinases that initiate downstream signaling via the PI3K/AKT or Ras/ERK pathways [87]. The physiological importance of IGF1R/IR hybrid receptors is still being investigated; however, it has been shown that hybrid receptors have a higher affinity for IGF-I and IGF-II compared to insulin and may shift cellular sensitivity away from insulin to these growth factors [87,88]. Increased hybrid receptor levels have consequently been associated with diseases such as diabetes mellitus [89] and cancer [88,90,91]. Dual silencing of IR and IGF1R abrogated the SOX9 response to IGF-II in our study while silencing each receptor alone had no effect. This further confirmed the relevance of the hybrid receptor for IGF-II-mediated fibrosis as our previous study reported that silencing the IGF1R/IR receptor reduced collagen and fibronectin levels in NL and SSc fibroblasts [17]. The hybrid receptor may be a potential therapeutic target for PF although there are several challenges associated with developing IGF1R/IR inhibitors, such as ensuring specific inhibition of the IGF1R/IR hybrid over the IR and IGF1R homodimers [87]. 

### 3.3. The IGF-II-SOX9 Pathway in Human Lung Fibroblasts

SOX9 is one of twenty SOX transcription factors characterized by a SRY-related HMG box sequence for DNA binding and gene regulation [30,31]. Similar to IGF-II, SOX9 regulates the development and growth of the lungs and other organs [92,93]. It is a master regulator of chondrogenesis, controlling several cartilage-associated ECM genes, such as collagen types II, IV, and aggrecan [94,95,96]. Our results indicated that SOX9 controls the induction of COL3A1, TGFβ2, and TGFβ3 downstream of IGF-II in human lung fibroblasts. The regulation of these genes by SOX9 has not been widely reported. Studies have focused on TGFβ1 regulation of SOX9 and/or SOX9 regulation of collagen types I and II in processes such as chondrogenesis, cancer, and other types of fibrosis [81,96,97,98,99,100,101,102,103]. In a murine model of cardiac hypertrophy, a pathology that involves fibrosis, *Sox9*, *Tgfb2*, and *Tgfb3* were mutually upregulated and predicted to interact; however, this was not experimentally confirmed or linked to ECM regulation [104,105]. Furthermore, *Sox9* deletion in murine models of renal and cardiac fibrosis did not identify differential expression of *Col3a1*, *Tgfb2*, or *Tgfb3* [79,80], which may be explained by differences between our models, including species, cell, and organ types. Conversely, our results indicated that SOX9 regulates *TGFβ2* and *TGFβ3,* and parallel findings in activated hepatic stellate cells (HSC) which exhibited downregulation of *Tgfb3* with *Sox9* knockdown [106]. Our findings were also validated in RNA sequencing data from SOX9-silenced IPF fibroblasts which showed a decrease in *COL3A1* and *TGFβ2* expression [36]. SOX9 is thus an important mediator of IGF-II-induced fibrosis, controlling levels of COL3A1, TGFβ2, and TGFβ3. 

The regulation of TGFβ2 and TGFβ3 by SOX9 has significant implications for the lung. *Tgfβ3* null mice have abnormal lung morphogenesis, a process that *SOX9* also regulates during development [92,107]. Furthermore, TGFβ2 and TGFβ3 promote PF [54]. Both genes were upregulated in IPF lung tissues compared to healthy controls [54,108], and SMAD-dependent luciferase activity assays suggested that the latent forms of TGFβ2 and TGFβ3 initiate profibrotic signaling without activation, unlike TGFβ1 [54]. TGFβ2 and TGFβ3 thus demonstrated distinct activation mechanisms compared to TGFβ1 during PF [54]. Furthermore, *Tgfb2* and *Tgfb3* knockdown in bleomycin-treated mice reduced collagen levels in the lung, implicating both factors in ECM deposition [54]. Therefore, IGF-II-SOX9-mediated regulation of TGFβ2 and TGFβ3 in our study suggests that SOX9 has a critical role in regulating and propagating PF downstream of IGF-II. 

In our study, SOX9 regulated *TGFβ2* and *TGFβ3* at the transcriptional level, while it regulated COL3A1 at the protein level. We considered that SOX9 may regulate collagenolytic enzymes to maintain COL3A1 levels. Cathepsins K, L, and B regulate ECM and their substrates include collagen types I, II, and III [109,110,111,112,113]. In our data, however, IGF-II downregulated expression of these cathepsins independent of SOX9. Instead, SOX9 mediation of P4HA2 may have accounted for reduced COL3A1 protein expression. As previously discussed, P4HA2 is involved in stabilizing collagen [32,55]. Reduced P4HA2 expression with SOX9 silencing may have prevented stabilization of COL3A1, resulting in degradation at the protein level. 

Several genes controlled by SOX9 in other fibroses were not controlled by SOX9 in our study. For example, SOX9 regulated *COL1A1* and *ACTA2* in IPF fibroblasts, murine cardiac tissues and fibroblasts, and activated rat HSCs. [36,79,106]. We did not observe an effect on these genes in our model. One possibility is that IGF-II regulates these targets via an unidentified factor other than SOX9. It is also plausible that SOX9 mediates these targets indirectly via TGFβ2 and TGFβ3. Alternatively, compensation by other SOX transcription factors may have contributed to our observation. There are multiple SOX transcription factors with functional redundancy, allowing one factor to compensate for the loss of another [30,31,114]. For example, SOX5/6/9 comprise a SOX-Trio that regulates genes involved in chondrogenesis [96], and while they function together, SOX5 and SOX6 (which belong to the same subfamily) can maintain chondrogenesis in single knockouts in mice [115]. Similarly, SOX9 and SOX10 are in the same subfamily and control similar processes during melanogenesis [29,31]. While these two factors have antagonistic roles in melanoma, whereby SOX10 promotes the cancerous phenotype and SOX9 reduces it [116], the high levels of SOX9 associated with metastasis can restore SOX10-mediated processes in the absence of SOX10 [117]. On this basis, we investigated SOX10 in our experiments; however, SOX10 levels were undetectable in fibroblasts. Therefore, if functional redundancy compensated for SOX9 loss, it was likely mediated by an alternative transcription factor. 

Silencing SOX9 in pulmonary fibroblasts demonstrated that SOX9 mediates fibrosis downstream of IGF-II. Therefore, SOX9 may be a potential target for treatment of pulmonary fibrosis. The role of SOX9 in cancer has prompted interest in developing SOX9 inhibitors, which could be leveraged for the treatment of PF [118,119,120].

We recognize that our study is limited to in vitro experimentation in normal lung fibroblasts and ex vivo experimentation in normal and SSc lung tissues. However, the use of human cells and tissues ensures direct relevance of our findings to the human disease, especially since IGF-II levels in mice decrease after birth and *Igf2* null mice are not viable [18,20,121], limiting the utility of mice in research focusing on IGF-II. We have identified several factors downstream of IGF-II in human lung fibroblasts that can be targeted for SSc-PF treatment. In addition, antibodies against IGF-II have been developed [122] and may have potential for PF treatment in view of the data showing that IGF-II tips the balance of fibrotic factors, extracellular matrix components, and enzymes responsible for ECM processing and degradation in favor of fibrosis. This further emphasizes the critical role of IGF-II in SSc-PF and the importance of inhibiting its activity to promote ECM homeostasis and reduce fibrosis.

## 4. Materials and Methods

### 4.1. In Vitro Primary Human Lung Fibroblast Cell Culture

Healthy human lung tissues were obtained from donors whose lungs were not used for transplantation under a protocol approved by the Medical University of South Carolina (MUSC) and the University of Pittsburgh Institutional Review Boards. Primary lung fibroblasts were isolated from the tissues and cultured as previously described [46] at 37 °C and 5% CO_2_ in DMEM (Cat#10-013, Corning, Corning, NY, USA) supplemented with 10% FBS (Cat#F4135, Millipore Sigma, Burlington, MA, USA) and 1% antibiotic/antimycotic (AB/AM) (Gibco by Thermo Fisher Scientific, Waltham, MA, USA). Fibroblasts in passages 3–8 were used for experiments. 

### 4.2. Ex Vivo Human Lung Tissue Culture and IGF-II Stimulation

Cores of equal size (5 mm) were prepared from healthy human lung tissues and cultured in serum free DMEM supplemented with 2% antibiotic/antimycotic (AB/AM) at 37 °C/5% CO_2_ under humidifying conditions. The cores were treated with 1X sterile phosphate buffered saline (PBS) (Cat#21-040-CV, Corning, Corning, NY, USA) as vehicle or 200 ng/mL recombinant human IGF-II (rhIGF-II) (Cat# 292-G2, R&D Systems, Minneapolis, MN, USA) for 24 h and 48 h. The cores were frozen at −80 °C for RNA and protein extraction. 

### 4.3. Donor Information 

Explanted lung tissues were obtained from control donors and patients with SSc as previously described [47]. The patient characteristics are summarized in Table S1. The demographic information for the healthy control donors and SSc patients whose lung tissues were used for steady-state SOX9 levels are described in Table S2. 

### 4.4. Culture and Treatment of Human Lung Fibroblasts 

Primary human lung fibroblasts were seeded in 35 mm dishes at 200,000 cells/dish in DMEM supplemented with 10% FBS and 1% AB/AM. Once confluent, the media was replaced with serum free DMEM, and the cells were incubated overnight. Fibroblasts were stimulated with vehicle/PBS (Cat#21-040-CV, Corning, Corning, NY, USA) or 200 ng/mL rhIGF-II (Cat# 292-G2, R&D Systems, Minneapolis, MN, USA) for the times indicated in the figure legends. RNA was harvested in 200 µL TRIzol reagent (Cat#15596018, Ambion by Life Technologies, Carlsbad, CA, USA) for qPCR analysis. 

### 4.5. Subcellullar Fractionation of Human Lung Fibroblasts

Primary human lung fibroblasts from different donors were seeded at 150,000 cells/well in 2 mL of DMEM supplemented with 10% FBS and 1% AB/AM media in 6-well cell culture treated plates. Once 70–90% confluent, the media was changed to serum free DMEM with 1% AB/AM for 12 to 24 h before stimulation with vehicle/PBS (Cat#21-040-CV, Corning, Corning, NY, USA) or 200 ng/mL rhIGF-II (Cat# 292-G2, R&D Systems, Minneapolis, MN, USA) for 2 and 4 h. Cells were harvested with trypsin (Cat#25-053-CI, Corning, Corning, NY, USA),and fractionations were performed using the subcellular protein fractionation kit for cultured cells (Cat# 78840, Thermo Fisher Scientific, Waltham, MA, USA) according to the manufacturer’s instructions. The subcellular fractions were supplemented with a protease inhibitor cocktail (included in the kit) and sodium orthovanadate (Cat#J60191, Alfa Aesar by Thermo Fisher Scientific, Haverhill, MA, USA).

### 4.6. Small Interfering RNA Transfection 

The IGF receptors–*IGF2R*, *IGF1R*, *IR*, and the *IGF1R/IR* hybrid–and *SOX9* were silenced in primary human lung fibroblasts from multiple donors using ON-TARGETplus small interfering RNA (Dharmacon, Lafayette, CO, USA). Fibroblasts were seeded at a density of 150,000 cells/well in 6-well plates with 2 mL of DMEM supplemented with 10% FBS and 1% AB/AM. At 70–90% confluency, the media was changed to 1 mL/well of AB/AM free DMEM with 10% FBS for transfection. Lipofectamine 2000 (Cat# 11668019, Invitrogen, Carlsbad, CA, USA) and siRNA reagents were mixed with OptiMEM 1X (Cat#51985-03P, Gibco by Thermo Fisher Scientific, Waltham, MA, USA) according to the manufacturer’s instructions and added to the cells. The siRNA reagents used for these experiments are listed in Table S3.

For IGF receptor silencing, 10 nM of siRNA was used per target. The wells for dual silencing of the *IGF1R/IR* hybrid receptor received 10 nM of siIGF1R and 10 nM of siIR for a final siRNA concentration of 20 nM. The wells that received siRNA for a single receptor (*IGF2R, IGF1R, IR*) were supplemented with 10 nM non-targeting siControl for a final siRNA concentration of 20 nM per transfection. The controls consisted of non-transfected cells and cells that were transfected with 20 nM of siControl. Following 48 h of silencing, the fibroblasts were serum starved for 2 h before stimulation with 200 ng/mL of rhIGF-II (R&D Systems) for 4 h. Whole cell lysates were harvested with RIPA lysis buffer supplemented with Halt^TM^ protease inhibitor single-use cocktail (Cat#78430, Thermo Fisher Scientific, Waltham, MA, USA) and sodium orthovanadate (Cat#J60191, Alfa Aesar by Thermo Fisher Scientific, Haverhill, MA, USA). 

For *SOX9* silencing, final concentrations of 20 nM non-targeting siControl and 20 nM of SOX9-specific siRNA were used. Cells were transfected for 24 h using Lipofectamine 2000 as described above. The media was then changed to serum free DMEM with 1% AB/AM. Twenty-four hours later, the cells were treated with vehicle/PBS or 200 ng/mL rhIGF-II in serum free DMEM with 1% AB/AM. Cells were harvested after 24 and 48 h of stimulation. RNA was harvested in 200 µL/well of TRIzol reagent (Cat#15596018, Ambion by Life Technologies, Carlsbad, CA, USA). Lysates were harvested after 24 h of stimulation in 200 µL/well of 2X SDS sample buffer and after 48 h of stimulation in 200 µL/well of RIPA buffer supplemented with Halt^TM^ protease inhibitor cocktail (Cat#78430, Thermo Fisher Scientific, Waltham, MA, USA) and sodium orthovanadate (Cat#J60191, Alfa Aesar by Thermo Fisher Scientific, Haverhill, MA, USA). Supernatants were centrifuged to remove debris and stored at −80 °C. 

### 4.7. RNA Extraction, cDNA, and qPCR

Ex vivo lung tissue cores were homogenized in TRIzol reagent using a bead ruptor apparatus (OMNI International, Kennesaw, GA, USA), and in vitro lung fibroblasts were scraped in TRIzol reagent (Cat#15596018, Ambion by Life Technologies, Carlsbad, CA, USA). RNA was extracted following the manufacturer’s instructions. The RNA was resuspended in UltraPure DNAse/RNAse-free distilled water (Cat# 10977015, Invitrogen, Carlsbad, CA, USA). A NanoDrop Lite spectrophotometer (Thermo Fisher Scientific, Waltham, MA, USA) was used to measure RNA concentrations. cDNA was synthesized from RNA on the C1000 Touch Thermal Cycler (Bio-Rad Laboratories, Hercules, CA, USA) using the Superscript IV First-Strand Synthesis kit (CAT# 18091200) and Oligo dT (12–18) primer (Cat#58862) from Thermo Fisher Scientific (Waltham, MA, USA) per the manufacturer’s protocol. For steady-state *SOX9* expression in normal and SSc lung tissues, 50 µM random hexamer primers (Invitrogen, Carlsbad, CA, USA) were used for cDNA synthesis per the manufacturer’s protocol.

Gene expression was measured using qPCR on an Applied Biosystems Step One Plus Real-Time PCR System (Thermo Fisher Scientific, Waltham, MA, USA). Gene expression was determined using the target gene 2^−ΔCT^ values normalized to *GAPDH* or *B2M.* All primers are listed in Table S4.

### 4.8. Immunoblotting

Ex vivo lung tissue punches were homogenized in RIPA lysis buffer supplemented with Halt^TM^ protease inhibitor cocktail (Cat#78430, Thermo Fisher Scientific, Waltham, MA, USA) and sodium orthovanadate (Cat#J60191, Alfa Aesar by Thermo Fisher Scientific, Haverhill, MA, USA). For steady-state *SOX9* expression in normal and SSc lung tissues, equal amounts of lung homogenates (25 µg/sample) were loaded for SDS-PAGE. In vitro lung fibroblast lysates were harvested in 2X SDS sample buffer or RIPA supplemented with Halt^TM^ protease inhibitor and sodium orthovanadate. Equal amounts of cellular lysates (RIPA), subcellular fractions, and supernatants were combined with 6X reducing SDS sample buffer. Samples were resolved using SDS-PAGE. Transfers were performed at 300 mA for 2 h onto nitrocellulose membranes (Cat# 10600015, Cytiva, Marlborough, MA, USA). The membranes were blocked in 5% nonfat dry milk (Nestle Carnation, Los Angeles, CA, USA) in Tris Buffered Saline + 0.05% Tween-20 (TBST) for 1 h at room temperature before incubation with primary antibody at 4 °C on a rotator for 16–24 h. The antibodies are listed in Table S5. The membranes were washed with TBST, incubated in secondary HRP-conjugated antibodies for 1 h at room temperature, washed with TBST, and imaged. The SignalFire Plus ECL reagent (Cat#12630S, Cell Signaling, Danvers, MA, USA) and the SuperSignal West Pico Plus reagent (Cat# 34578, Thermo Fisher Scientific, Waltham, MA, USA) were used for imaging with the iBright (Thermo Fisher Scientific) or FluorChem R (ProteinSimple, San Jose, CA, USA) imaging systems. 

Densitometric analysis was carried out with ImageJ software version 1.53k [123]. Lysates and fibroblast cytoplasmic fractions were normalized to the housekeeping protein GAPDH, while cellular supernatants and nuclear fractions were normalized to their total protein concentrations. Nuclear and chromatin fraction concentrations were measured on a NanoDrop Lite Spectrophotometer (1A/cm = 1 mg/mL) (Thermo Fisher Scientific, Waltham, MA, USA). Equivalent loading of nuclear extracts was also confirmed by reprobing membranes with anti-TBP antibody. Supernatant concentrations were measured using the Pierce Detergent Compatible Bradford Assay Kit (Cat#23246, Thermo Fisher Scientific, Waltham, MA, USA), and equal loading was visualized using the No-Stain Protein Labeling Reagent kit (Cat#A44449, Thermo Fisher Scientific, Waltham, MA, USA) or the Ponceau S stain (Cat#628, Allied Chemical, Morristown, NJ, USA). 

### 4.9. ELISA

Commercially available ELISA kits were used to measure TGFβ2 (Cat#ELH-TGFb2-1, RayBiotech, Norcross, GA, USA) and TGFβ3 (Cat#EA100646, Origene, Rockville, MD, USA) levels in lung fibroblast supernatants. Samples were measured in duplicate, and latent TGFβ2 and TGFβ3 were activated to the immunoreactive forms according to the manufacturer’s protocol.

### 4.10. Statistical Analysis

Statistical analyses were performed with SAS version 9.4 (SAS Institute, Cary, NC, USA). Raw values (qPCR 2^−ΔCT^, immunoblot densitometry units, or protein concentrations) were plotted as mean ± SEM. Student’s paired *t*-tests for paired samples and two-sample *t*-test independent samples were performed for experiments including two groups. Experiments including multiple groups were analyzed using repeated measures one-way or two-way ANOVA with Tukey’s post-hoc test. Statistical assumptions for ANOVA models were checked graphically and log-transformations were applied when needed to improve model assumptions. *p*-values below 0.05 were considered significant. Graphs were prepared in GraphPad Prism version 9 (GraphPad Software, San Diego, CA, USA).

## 5. Conclusions

Our study emphasizes the significance of IGF-II as a profibrotic signaling molecule in human lung and elucidates a pathway involving SOX9 by which IGF-II induces fibrosis in lung fibroblasts. IGF-II increases profibrotic signaling via TGFβ2/TGFβ3 upregulation, increases collagen biosynthesis and crosslinking through COL3A1, P4HA2, P4Hβ, P3H2, LOXL2, LOXL4 upregulation, and decreases collagen degradation by downregulating CTSB, CTSK, CTSL (Graphical Abstract). In addition, IGF-II engages the transcription factor SOX9 via the IGF1R/IR hybrid receptor to regulate TGFβ2, TGFβ3, COL3A1, and P4HA2, while other targets are SOX9-independent (Graphical Abstract). Our study broadens our understanding of the mechanisms mediating pulmonary fibrosis and identifies new targets for the development of therapies targeting the IGF-II axis.

## Data Availability

All data is contained within the article or supplementary material.

## Supplementary Materials

The following supporting information can be downloaded at: https://www.mdpi.com/article/10.3390/ijms241411234/s1.

## Abbreviations

ACTA2, actin alpha 2 smooth muscle; COL1A1, collagen type I alpha 1 chain; COL3A1, collagen type III alpha 1 chain; CTGF, connective tissue growth factor; CTSB, cathepsin B; CTSK, cathepsin K; CTSL, cathepsin L; ECM, extracellular matrix; IGF-I, insulin-like growth factor I; IGF-II, insulin-like growth factor II; IGFBP, IGF binding protein; IGF1R, IGF-1 receptor; IGF-2R, IGF-II receptor; IR, insulin receptor; IGF1R/IR, hybrid IGF-1R and IR receptor; IPF, idiopathic pulmonary fibrosis; LOX, lysyl oxidase; LOXL2, lysyl oxidase like 2; LOXL4, lysyl oxidase like 4; MMP, matrix metalloproteinase; NL, normal lung; P3H2, prolyl 3-hydroxylase 2; P4HA2, prolyl 4-hydroxylase subunit alpha 2; P4Hβ, prolyl 4-hydroxylase subunit beta; PF, pulmonary fibrosis; PTM, posttranslational modification; SOX9, SRY-box transcription factor 9; SSc, systemic sclerosis; TGFβ2, transforming growth factor beta 2; TGFβ3, transforming growth factor beta 3; TIMP, tissue inhibitors of metalloproteinases.
