# Supplementary material for: The Role of SOX9 in IGF-II-Mediated Pulmonary Fibrosis
Source: Int J Mol Sci. 2023 Jul 8;24(14):11234. doi: 10.3390/ijms241411234 (PMC10378869; doi:10.3390/ijms241411234)
Supplement: Supplementary file 1 [file ijms-24-11234-s001.zip › ijms-2330099-supplementary.pdf]

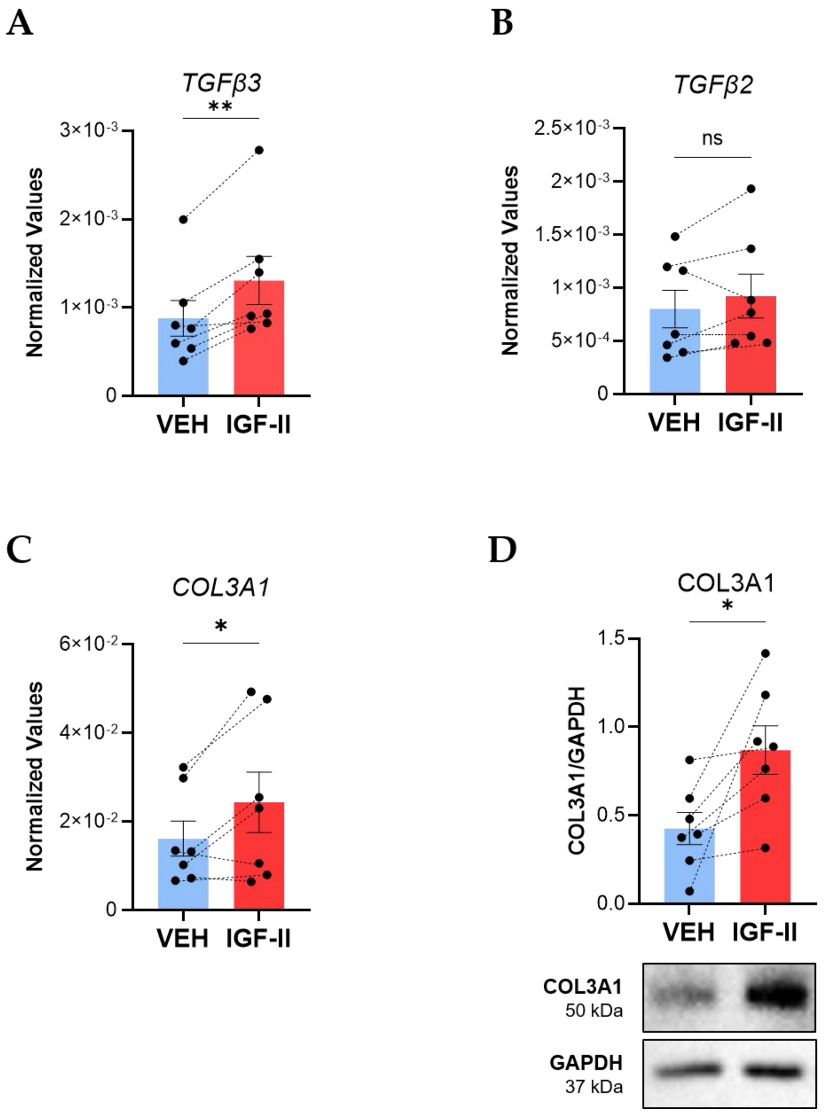

Figure S1: IGF-II promotes fibrosis in normal human lung (NL) tissue explants from multiple donors. (A) Transcript levels of *TGFβ3* in ex vivo NL tissue explants treated with vehicle or IGF-II for 48h (n=7). **(B)** Transcript levels of *TGFβ2* in NL tissue explants as described for Panel A (n=7). **(C)** Transcript levels of *COL3A1* in NL tissue explants treated for 24h (n=7). **(D)** COL3A1 levels in NL tissue homogenates after 24h stimulation (n=7). The dashed lines indicate pairing between cell lines from the same donor. Raw $2^{-\Delta CT}$ values are graphed for qPCR data after normalization to the housekeeping gene *B2M*. Raw densitometry values determined by ImageJ software after normalization to GAPDH are plotted for immunoblot quantification. Paired Student's *t*-Test, ns = not significant, * $p \leq 0.05$, ** $p \leq 0.01$. Mean $\pm$ SEM.

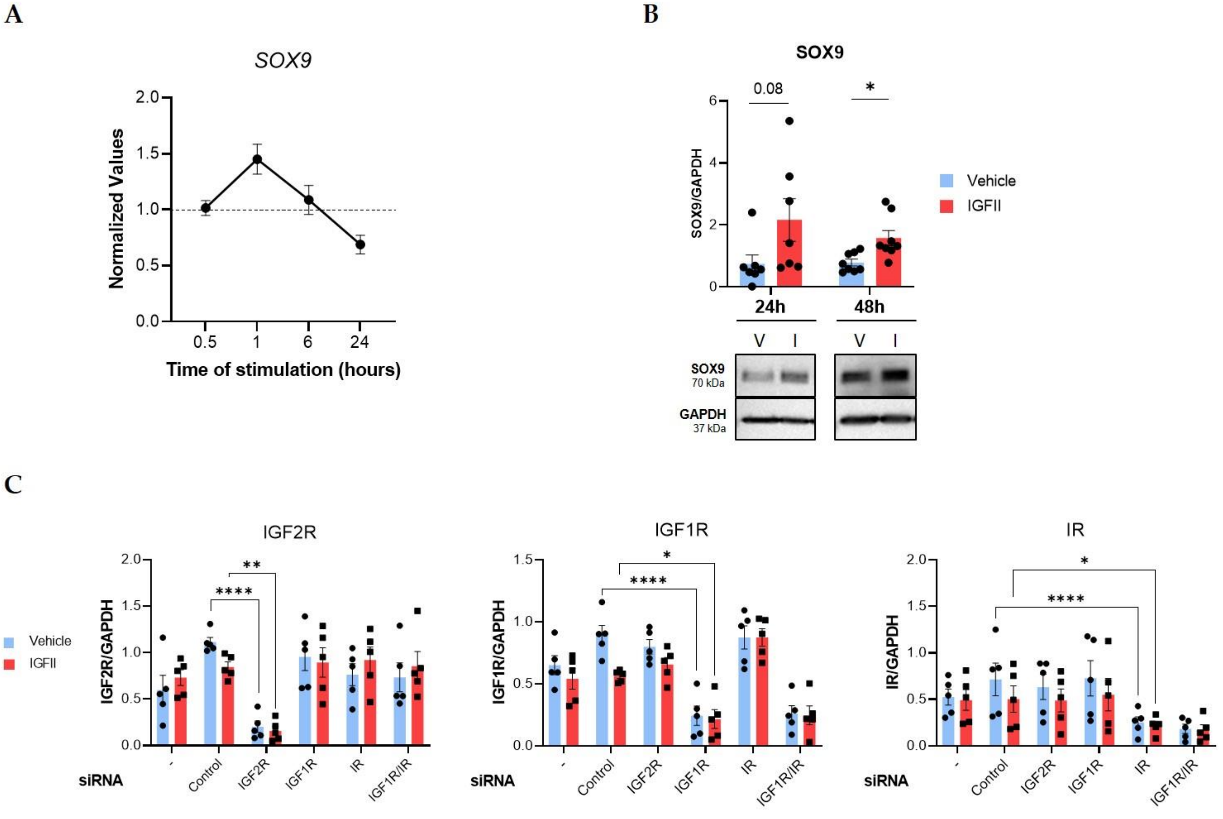

Figure S2: NL fibroblasts were stimulated with recombinant human IGF-II (200 ng/mL) or vehicle control (PBS) and SOX9 levels were measured. **(A)** qPCR analysis of *SOX9* levels normalized to *B2M* in fibroblasts treated with IGF-II for multiple timepoints: 0.5h, 1h, 6h, and 24h (n=6). The vehicle levels are set to 1 and shown in the dashed line. Mean ± SEM graphed for IGF-II. Two-Way ANOVA: 1h, $p = 0.094$; 24h, $p = 0.055$. **(B)** Representative immunoblots and quantification of SOX9 in lysates after 24h (n=7) and 48h (n=8) of IGF-II stimulation in human NL fibroblasts. Raw densitometry values measured by Image J software and normalized to GAPDH. Paired Student's *t*-Test **(C)** The IGF system receptors – IGF2R, IGF1R, IR, and hybrid IGF1R/IR – were silenced in lung fibroblasts before stimulation with vehicle/PBS and IGF-II for 4h (n=5). Immunoblot quantification of receptor levels to confirm transfection efficiency. Representative immunoblots shown in Figure 2E. Data normalized to GAPDH loading control. (-): Non-transfected control. V: vehicle/PBS; I: IGF-II. Repeated Measures Two-way ANOVA with Bonferroni correction for 2 pairwise comparisons, * $p \leq 0.05$, ** $p \leq 0.01$, **** $p \leq 0.0001$. Mean ± SEM.

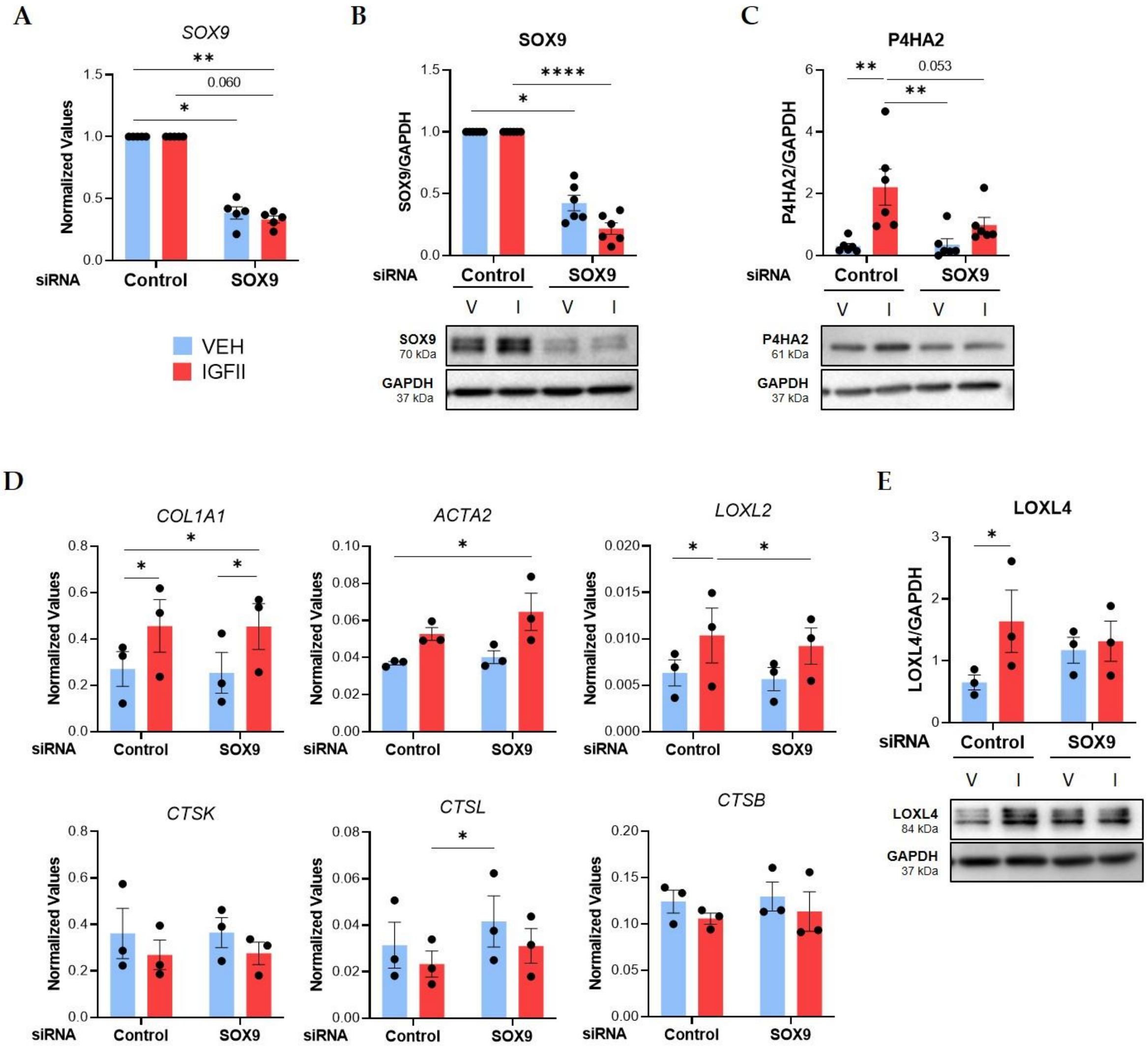

Figure S3: Normal human lung fibroblasts from different donors were transfected with non-targeting control or SOX9-specific siRNA before stimulation with vehicle/PBS (V) and IGF-II (I) for 24 or 48 hours **(A)** qPCR analysis of SOX9 transcripts in 24h samples (n=5). **(B)** SOX9 protein levels and representative immunoblot in 48h lysates (n=6). Fold change values are graphed with the results of repeated measures ANOVA between the normalized raw values of control and SOX9-silenced samples respective to treatment. **(C)** Protein levels of P4HA2 in 48h lysates (n=6). **(D)** Transcript levels of SOX9-independent targets in 24h samples (n=3). **(E)** Protein levels of LOXL4 in 48h lysates (n=3). Raw $2^{-\Delta CT}$ values are graphed for qPCR data after normalization to the housekeeping gene (*GAPDH* or *B2M*). Raw densitometry values determined by ImageJ software are plotted for immunoblot quantification. Repeated Measures ANOVA with Tukey post hoc test, * $p \leq 0.05$, ** $p \leq 0.01$, **** $p \leq 0.0001$. Mean ± SEM.

Table S1: SSc patient characteristics: EA, European American; AA, African American; PF, pulmonary fibrosis; PAH, pulmonary arterial hypertension; NA, not available.

Donor Code	Age (years)	Sex	Ethnicity	Diagnosis	Smoker
SSc-8	26	Female	EA	PF	No
SSc-11	45	Male	EA	PF	No
SSc-19	37	Female	EA	PF	No
SSc-23	51	Female	EA	PF	Yes
SSc-24	45	Male	EA	PF	No
SSc-26	57	Male	EA	PF	No
SSc-27	41	Female	EA	PF	Yes
SSc-81	67	Male	EA	PF + PAH	NA
SSc-82	54	Female	EA	PF + PAH	No
SSc-85	62	Female	AA	PF + PAH	Yes
SSc-87	64	Male	EA	PF	Yes
SSc-110	50	Male	EA	PF + PAH	No

Table S2: Donor characteristics for normal lung (NL) and Scleroderma (SSc) lung tissue cores used to quantify baseline SOX9 levels.

Characteristics	NL	SSc
n	10	12
Age (Average $\pm$ SEM)	44.6 $\pm$ 2.3	49.9 $\pm$ 3.3
Male	5	6
Female	4	6
NA	1	-
EA	6	11
AA	-	1
NA	4	-

Table S3: List of small interfering RNA (siRNA) reagents used in experiments.

siRNA Target	Catalog Number	Species	Company
IGF1R	L-003012-00-0005	Human	Dharmacon by Horizon
IGF2R	L-010601-00-0005	Human	Dharmacon by Horizon
INSR	L-003014-00-0005	Human	Dharmacon by Horizon
SOX9	L-021507-00-0005	Human	Dharmacon by Horizon
On-Target Plus Non-Targeting Pool (Control)	D-001810-10-20	Human	Dharmacon by Horizon

Table S4: List of primers purchased from Thermo Fisher Scientific for qPCR.

Primer Target	Catalog Number	Species	Reporter
ACTA2	Hs00426835_g1	Human	FAM
B2M	Hs00187842_ml	Human	VIC
COL1A1	Hs00164004_ml	Human	FAM
COL3A1	Hs00943809_m1	Human	FAM
CTSB	Hs00947433_m1	Human	FAM
CTSK	Hs00166156_m1	Human	FAM
CTSL	Hs00964650_m1	Human	FAM
GAPDH	Hs02758991_g1	Human	FAM
LOX	Hs00184700_m1	Human	FAM
LOXL2	Hs00158757_m1	Human	FAM
P3H2	Hs00216998_m1	Human	FAM
P4HA2	Hs00990001_m1	Human	FAM
SOX9	Hs00165814_m1	Human	FAM
TGFβ2	Hs00234244_m1	Human	FAM
TGFβ3	Hs01086000_m1	Human	FAM

Table S5: List of antibodies used for immunoblotting.

Target	Company	Catalog Number	Primary Dilution	Secondary Dilution
Amersham ECL Rabbit IgG, HRP-linked whole Ab (from donkey)	Cytiva	NA934	-	-
Anti-goat IgG HRP	Santa Cruz	SC-2354	-	-
Anti-Mouse IgG (H+L), HRP Conjugate	Promega	W4021	-	-
Cathepsin K	Santa Cruz	SC-48353	1:1000	1:5000
COL3A1 (S-17)-R	Santa Cruz	SC-8780-R	1:1000	1:1000
GAPDH	Santa Cruz	SC-47724	1:5000	1:10,000
IGF1Rβ	Cell Signaling	3027	1:1000	1:5000
IGF2R	Cell Signaling	15128	1:1000	1:5000
IRβ	Cell Signaling	23413	1:1000	1:5000
LOXL4	Santa Cruz	SC-48731	1:1000	1:5000
P4HA2	Novus	NB110-40494	1:1000	1:5000
P4HB	Chemicon	MAB2701	1:1000	1:5000
SOX9	Cell Signaling	82630	1:1000	1:5000
TBP	Abcam	ab51841	1:1000	1:5000
